# Our Size

**Published:** 1877-03-20

**Authors:** 


OUR SIZE.—A subscriber, while much pleased with the matter of our new journal, thinks "the size has been cut off more than the price." Our friend is mistaken. The double-column page with increased length and width of form contains a much larger amount of reading matter and less waste of margin, so that in point of fact our cut off in size corresponds with the reduction in price. It is our intention also, as soon as our list will justify the outlay, to make the type smaller, and thus increase the amount of reading matter.

